# Prediction of Protein Sites and Physicochemical Properties Related to Functional Specificity

**DOI:** 10.3390/bioengineering8120201

**Published:** 2021-12-03

**Authors:** Florencio Pazos

**Affiliations:** Computational Systems Biology Group, Systems Biology Department, National Centre for Biotechnology (CNB-CSIC), c/Darwin, 3, 28049 Madrid, Spain; pazos@cnb.csic.es

**Keywords:** protein family, protein multiple sequence alignment, protein function, protein specificity-determining position

## Abstract

Specificity Determining Positions (SDPs) are protein sites responsible for functional specificity within a family of homologous proteins. These positions are extracted from a family’s multiple sequence alignment and complement the fully conserved positions as predictors of functional sites. SDP analysis is now routinely used for locating these specificity-related sites in families of proteins of biomedical or biotechnological interest with the aim of mutating them to switch specificities or design new ones. There are many different approaches for detecting these positions in multiple sequence alignments. Nevertheless, existing methods report the potential SDP positions but they do not provide any clue on the physicochemical basis behind the functional specificity, which has to be inferred a-posteriori by manually inspecting these positions in the alignment. In this work, a new methodology is presented that, concomitantly with the detection of the SDPs, automatically provides information on the amino-acid physicochemical properties more related to the change in specificity. This new method is applied to two different multiple sequence alignments of homologous of the well-studied RasH protein representing different cases of functional specificity and the results discussed in detail.

## 1. Introduction

Proteins are behind most cellular process that sustain life. The protein repertory coded by a given organism can be obtained by sequencing its genome, which is relatively easy and cheap today due to the recent improvements in sequencing technologies [1]. Metagenomics projects are also increasing the number of sequenced proteins and protein fragments [2]. As a consequence, the number of known protein amino-acid sequences deposited in public databases continues to grow. Bioinformatics approaches have been developed to distill useful information from these large amounts of data [3], complementing the more expensive and time-consuming experimental approaches.

These in-silico approaches, which take advantage of this deluge of sequence information, allow, among other things, to predict a protein’s function and its functional/active sites, structural features including its three-dimensional structure, interactions with other proteins and small molecules, etc. The base of most of these approaches is to use sequence comparison methods to retrieve similar proteins from databases, to subsequently align and compare them in “multiple sequence alignments” (MSAs) [4]. In a MSA, the aminoacid sequences of homologous proteins (i.e., those sharing a common ancestor) are placed in a way that potentially evolutionary equivalent residues stack together (in the same column in the most common representation) in such a way that they can be compared. Homologous proteins share the same overall three-dimensional structure [5] and many functional features [6], inherited from their common ancestor. As a column of a MSA (termed “position”) can be regarded as a representation of the amino-acid changes allowed by the evolution at a particular site in a set of homologous proteins, a lot of information related to the protein’s structure and function can be extracted by studying the patterns of amino-acid variation of its positions [7]. The most obvious variation pattern related to functionality is complete conservation, and as such it was used for predicting protein functional sites since long ago [8] and methods continue to be developed [9,10]. A position where evolution did not allow any change must have some functional or structural importance for the protein.

Another positional pattern indicative of functional importance is the so-called “family-dependent conservation”. When the set of proteins within a MSA can be divided into sub-families according with some functional criteria (e.g., sub-groups with slightly different functional specificities within the global function shared by all the homologs), positions that are differentially conserved within the subfamilies show up. For example, a family of enzymes that catalyze the same chemical transformation (e.g., dehydrogenation) could be split into subfamilies according with the particular substrate where that transformation is applied (e.g., malate vs. lactate dehydrogenases). That substrate specificity will be reflected in the aminoacid patterns of particular positions involved in binding the substrate: they will tend to be conserved due to their importance in binding, but with a different amino-acid in each subgroup to accommodate its particular substrate. On the other hand, positions involved in the common function (dehydrogenation) would tend to be fully conserved. Those positions related to functional specificity are termed “specificity-determining positions” (SDPs) [7], and they complement those fully conserved as predictors of functionality, as both reflect different functional/evolutionary constraints.

SDP analysis is routinely used for detecting these positions related to functional specificity, in many cases followed by experimental work aimed at switching or changing that specificity by mutating these positions (see examples in [11]), which has obvious applications not only in basic research, but in biotechnology and biomedicine as well. There are many different approaches for detecting SDPs in MSAs [7,12,13,14,15,16,17]. Depending on whether the subfamily classification from which SDPs are going to be extracted is that implicit in the sequence relationships represented by the MSA or is external to it, these approaches are classified in “unsupervised” and “supervised” methods, respectively [18]. In the first case, the only required input is the MSA itself, as proteins subgroups are inferred, in one way or another, from it. In the second case, apart from the MSA, the method requires as additional input a functional classification of the proteins. Most methods belong to the first class as in most cases, in agreement with a scenario of “divergent evolution”, the protein classification implicit in the MSA (i.e., phylogeny) recapitulates the functional classification. But in some particular cases, or for some particular functional features of the many a family is subject to, there is a “function-phylogeny” disagreement and supervised approaches are required (e.g., [18,19,20,21]).

A family of approaches for detecting SDPs, globally termed “mutational behavior-based methods”, is based on comparing a matrix representing the pairwise similarities of the proteins within the MSA, with an equivalent matrix encoding the amino-acid pairwise similarities for a particular position. The idea is that positions with the “SPD behavior” described above would have a matrix of amino-acid similarities resembling that of the proteins, as subgroups of similar proteins would match the same (or similar) amino-acid and the other way around [22,23,24]. This approach can be turned into “supervised” by simply substituting the matrix with the protein pairwise similarities by an external matrix quantifying the proteins’ functional similarities one wants to impose [18].

All methods for detecting SDPs report the positions potentially involved in controlling functional specificity but they do not provide any clue on the physicochemical basis of that specificity. That should be done a-posteriori by manually inspecting the reported positions and the amino-acid changes there. This, besides not being feasible for large collections of MSAs, is limited by the user knowledge on amino-acid properties. It would be desirable a method that automatically reports not only the differentially conserved positions but also the physicochemical property(s) more related to those differences, as they would provide a physicochemical explanation for the specificity shift.

In this work, I present a method that automatically extracts SDPs from MSAs and concomitantly generates a rank of the amino-acid physicochemical properties more related to these positions. The method can work both, in “supervised” and “unsupervised” mode.

## 2. Materials and Methods

**Figure 1:**
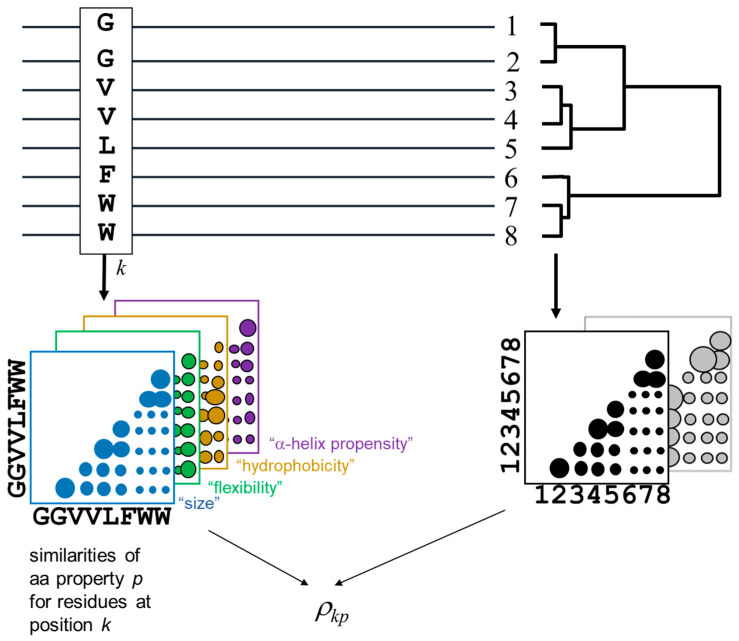
Schema of the methodology. For a given position (column) of the MSA, different matrices of inter-aminoacid similarities of physicochemical properties are derived (colored matrices). These are compared with the equivalent matrix containing the protein pairwise similarities (right) in order to detect positions and physicochemical properties whose change pattern at that position reflect the MSA phylogeny. Instead of a matrix of protein sequence similarities, a generic matrix with “functional similarities” can be imposed (grey), to apply the methodology in “supervised” mode.

The methodology for detecting the positions of a MSA and the corresponding amino-acid physicochemical properties more related with the MSA phylogeny is outlined in Figure 1. The method is derived from [18,22].

The phylogenetic relationships within the alignment are represented by a distance matrix containing the pairwise similarities between the proteins in the MSA (bottom right in Figure 1). For a given pair of proteins, the value of the corresponding entry in this matrix is the average residue similarity between them, extracted from the McLachlan aminoacid substitution matrix [25]. The subfamily composition of the MSA (i.e., clades in the implicit phylogenetic tree) would be reflected as clusters in this matrix. To impose an external functional classification of the proteins (“supervised” mode), an arbitrary external matrix quantifying pairwise functional similarities can be used instead.

For a given position of the MSA, an equivalent matrix is built containing the pairwise residue similarities for the amino-acids within that positions according with the same McLachlan substitution matrix. Positions with a family-dependent conservation pattern (SDPs) are expected to present a position matrix similar to the global matrix of the MSA (previous paragraph) [22]. To detect amino-acid physicochemical properties related to the family composition, similar matrices are constructed for the position but containing pair-wise similarities of these particular physicochemical properties (colored matrices in Figure 1), instead of the overall amino-acid similarities reflected by a substitution matrix.

The similarities between these position matrices and the MSA matrix is quantified using a Spearman rank-order correlation coefficient [26]. For a given position *k* and a physicochemical property *p*, we obtain its SDP score as:

$$\rho_{kp}=\frac{\sum_{i,j}\left(S_{ijkp}^{\prime }-\bar{S^{\prime }})\cdot (M_{ij}^{\prime }-\bar{M^{\prime }}\right)}{\sqrt{\sum_{i,j}{\left(S_{ijkp}^{\prime }-\bar{S^{\prime }}\right)}^{2}}\cdot \sqrt{\sum_{i,j}{\left(M_{ij}^{\prime }-\bar{M^{\prime }}\right)}^{2}}}$$

where the sums run for all pairs of proteins (*i, j*). *S_ijkp_* is the similarity between the value of the physicochemical property *p* of the residue *k* in protein *i* and that of property *p* of residue *k* in protein *j*. This value is set to 0 for any pair involving a gap. For a given pair (*m* and *n*) of the 20 natural amino-acids, their similarity of physicochemical property *p* is calculated as:

*S*_*pnm*_ = *MAXdiff*_*p*_ − |*S*_*pn*_ − *S*_*pm*_|

where *S_pn_* and *S_pm_* are the original values of the physicochemical property *p* for amino-acids *m* and *n*, and *MAXdiff_p_* is the maximum difference of that property for all pairs between the 20 natural aminoacids.

*M_ij_* is the overall sequence similarity between proteins *i* and *j* when working in “unsupervised mode”, or the (externally imposed) functional similarity between proteins *i* and *j* when working in “supervised” mode.

*S’* and *M’* are the ranked values of *S* and *M*, respectively (ties being assigned mid-ranks [26]). $\bar{S^{\prime }}$ and $\bar{M^{\prime }}$ are the corresponding average values of these ranked matrices. Positions with more than 10% gaps are excluded from these calculations.

Consequently, this *ρ_kp_* score is high when, for the residue pattern of position *k*, the matrix of pairwise similarities of chemical property *p* resembles the matrix with the overall protein similarities (or that with functional similarities—supervised mode).

### 2.1. Scales of Amino-Acid Physicochemical Properties

The method can incorporate any table associating quantitative values to each of the 20 natural amino-acids, in any scale as the rank-correlation criteria naturally re-scales the values.

In this particular work, we used 12 amino-acid properties downloaded from the ProtScale server [27]: “relative mutability” (relative mutability of the 20 amino-acids relative to Ala), “polarity/Grantham” (amino-acid polarity), “hydrophobicity/Kyte&Doolittle”, “hydrophobicity/Eisenberg” and “hydrophobicity/Chothia” (three different scales of amino-acid hydrophobicity, some are experimental while others are derived from database information, such as the proportion of buried vs. exposed residues), “average flexibility” (a flexibility index calculated from the fluctuational amplitudes of the amino acid), “coil/Deleage&Roux” (proportion of residues neither in alpha nor in beta conformation in protein structures), “molecular weight” (molecular mass of each amino-acid, in Daltons), “bulkiness” (a measure of the volume of the amino-acids), “beta-sheet/Chou&Fasman”, “alpha-helix/Deleage&Roux” and “beta-turn/Levitt” (proportion of residues in these three secondary structure states). More information on these scales can be obtained from the references given in the ProtScale server [27].

We also used the five “main factors” generated by [28]. In that work, a dimensionality reduction technique was applied to around 500 amino-acid properties in order to extract the main factors (combinations of these properties) separating the 20 aminoacids. The authors came to an interpretation for their five main factors according to the properties that contribute more to them (factor1: polarity, factor2: secondary structure, factor3: molecular size/volume, factor4: number of codons, factor5: electrostatic charge), and provide the values of these factors for the 20 amino-acids.

### 2.2. Test Examples

We used the well-characterized Ras superfamily of proteins to test the method.

On one hand, we generated a MSA with 97 homologs of the RasH oncogene. This MSA contains homologs of this protein in the Uniprot database down to 30% sequence identity and was filtered for redundancy at 95%. The alignment was generated with ClustalW (default parameters). It comprises different Ras families with different functional specificities (i.e., binding different effectors) within the common function for all of them (GTP binding and hydrolysis). These functional subfamilies are well reflected in the phylogeny and consequently this constitutes a good example for testing the method in “unsupervised mode”.

We generated a second alignment with structural analogs of the same RasH protein. We started from the structural alignment automatically generated by Dali [29] from the three-dimensional structure of the Ras oncogene (PDBid: 1ctqA). This alignment contains proteins of known structure binding different ligands, such as nucleotides (GTP, FMN, FAD, etc.), nucleosides, and sugars. The alignment was filtered, leaving only chains with a structural similarity with the master (1ctqA) higher than 6.0 (Dali’s Z-score), removing redundancy above 40% sequence identity, and removing structures without bound ligand. The final alignment contains 24 proteins binding different ligands. In this case, there is a disagreement between the classification of the proteins implicit in this alignment and the functional classification (according to the ligand they bind) mainly due to the fact that the structural alignment of these distant proteins (due to the redundancy cutoff imposed) is relating remote homologs at very high distances [18], and hence it constitutes a good example to test the method in “supervised mode”. For that, as a measure of functional similarity between two proteins, we took the chemical similarity between the ligands they bind, measured as the Tanimoto coefficient [30]. For pairs where one of the proteins is binding more than one ligand, we took the pairs of ligands with highest similarity. Solvent and non-functional small molecules are excluded. All the guanine nucleotides (e.g., GTP, GDP, GNP) are considered as a single class (‘GXP’) since the same protein is able to bind all of them, irrespective of the fact that in a particular structure it is co-crystallized with one or another. So, for example, two proteins binding GTP and GDP, respectively, are considered as 100% functionally identical.

## 3. Results and Discussion

**Figure 2:**
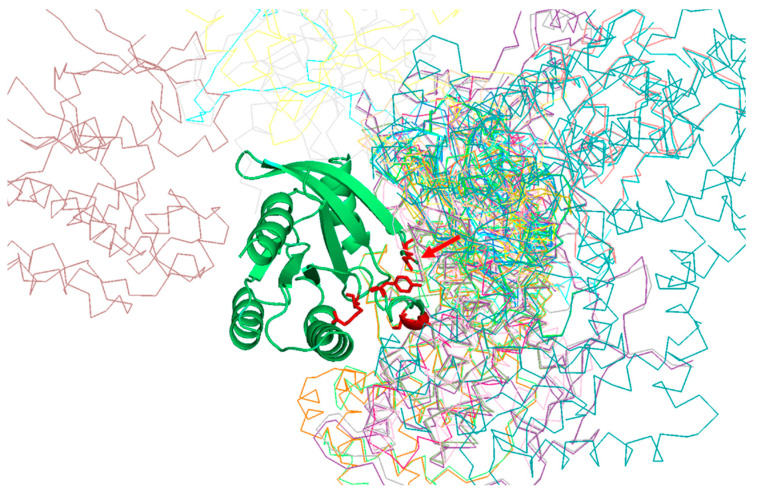
Predicted SDPs for the MSA of RasH homologs. The figure shows the structural superimposition of all complexes available for RasH in the RCSB structural database. Take a look at [32] for details on the generation of this structural superimposition. The 3D structure of RasH is shown in green and ribbon representation. The five SDPs with highest scores are shown in red and sticks representation. The arrow marks the position of residue nr. 37.

Table 1 shows the positions of the RasH MSA with highest SDP scores, as well as the corresponding physicochemical properties. The five residues whose physicochemical properties correlate more with RasH phylogeny (score ≥ 0.67) are 37, 73, 65, 66, and 71. All these resides are in the structural region of RasH involved in most of the interactions of this family of proteins with their different effectors [31] (Figure 2).

**Figure 3:**
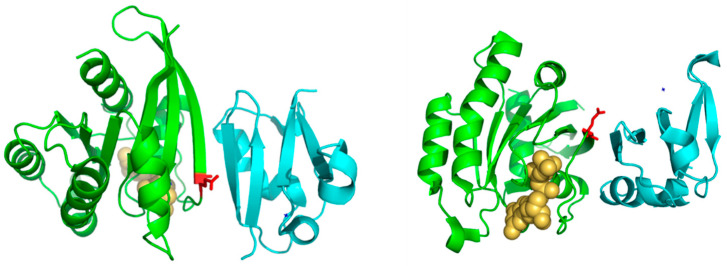
Detailed view of a RasH homolog complex marking residue 37. The two panels show two views of the complex between RasH homolog Rap1A (green) and the Ras-binding domain of the serine/threonine kinase c-Raf1 (blue) (PDBid: 1C1Y). The residue corresponding to position nr. 37 is shown in red. The GTP analog bound to Rap1A is shown in yellow and spacefill representation.

Of particular interest is residue 37 (Figure 2), which has the highest score both, based on an amino-acid substitution matrix and on many physicochemical properties (Table 1). This position consistently shows up as the “best” SDP for the Ras superfamily using different SDP-detection approaches as well as visual inspection of the MSA [31,33,34], as it has a clear family-dependent conservation pattern: i.e., conserved Glu in Ras, Ala in Ral, Phe in Rho, and Gly in Rab. Indeed, the crucial role of this position in controlling binding specificity has been experimentally demonstrated, and its single mutation in Ras to the equivalent Ral residue (E37A) is enough to significantly switch the interaction specificity of Ras making it able to interact with Ral effectors [31]. In spite of the many publications discussing the important role of this position for controlling specificity, its physicochemical bases are largely unknown (i.e., why Ras needs a Glu at that position to interact with its effectors, Ral and Ala, etc.). Among the 17 physicochemical properties and “factors” explored for the amino-acids at position 37, that more correlated with the family phylogeny is “beta propensity” (Table 1). Position 37 corresponds to the first residue of a beta-strand in the 3D structure of Rap1A (Figure 3). Consequently, residues with different propensities to form beta-strands at this position would affect the length and properties of the extreme of that beta-strand. It is known that the structural basis of the interaction between Ras proteins and many of their effectors is the formation of an inter-molecular antiparallel beta-sheet between the two proteins [35], as illustrated by the complex between Rap1A and the Ras binding domain of Raf1 kinase (Figure 3). Consequently, changes in the length and other characteristics of the beta-strands involved in that interaction could be important for controlling the specificity. In the complex between RalA and Sec5 [35], the fully conserved Ala of Ral at this position 37 (A48 in Ral) is involved in backbone hydrogen bonds with Sec5, contributing to the stability of the inter-molecular beta-sheet. This Ala is fully conserved and, as commented above, its change can switch the Ras/Ral interactions specificity, in spite of its side chain not being involved in interactions in this particular complex. Thus, maybe it is not (directly) its side chain that is important but its propensity to form beta-strand at that particular position.

To illustrate the application of the method in a “supervised” way, we used it to locate the residues and their physicochemical properties responsible for the small-molecule binding specificity in a MSA of Ras structural homologs, using as metric of “functional similarity” between proteins the chemical similarity between the ligands they bind (see Methods).

**Figure 4:**
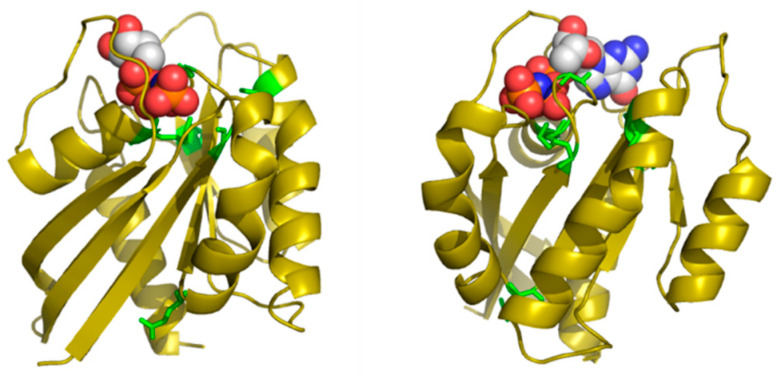
Predicted SDPs for the MSA of RasH structural homologs. The two panels show two views of the 3D structure of RasH in complex with a non-hydrolysable GTP analog (PDBId 1CTQ). The SDPs are shown in green. The GTP analog is shown in spacefill representation and colored by atom type to highlight the polar/charged phosphate groups.

Table 2 shows the positions of this MSA with highest SDP scores, as well as the corresponding physicochemical properties. The six residues whose physicochemical properties correlate more with RasH phylogeny (score ≥ 0.50) are 16, 10, 76, 13, 115, and 89. Most of these positions cluster around the ligand binding site of this set of structural homologs (Figure 4), with the exception of E76, which is far apart from that site. Indeed, residues G10, G13 and K16 form part of the well-known G1/P-loop motif (**G**AG**G**VG**K**S) characteristic of nucleotide-binding proteins [34], and they are close to the phosphate groups of the GNP bound to the particular structure of RasH shown in Figure 4. Regarding the physicochemical properties of the residues within these positions (Table 2), most of them are related to hydrophobicity/polarity and charge (including “factor1” and “factor5”—see Methods).

In this particular MSA, the functional specificity is related to the binding of different small-molecule ligands within the structurally-equivalent pocket of all these remote homologs with the same 3D structure (see Methods). Consequently, the method correctly identifies positions around this binding site. Most of the ligands bound to these 24 proteins have a nucleotide/nucleoside-like common framework and a more variable part such as the phosphates of the ATP. Three out of the five SDPs that are pointing to the binding site are indeed marking this more variable part, indicating that it is there where specificity resides. The physicochemical basis of that binding specificity predicted by the method (hydrophobicity/polarity and charge) is also in agreement with these main differences between ligands: i.e., highly polar/charged phosphates of ATP vs. less polar/uncharged molecules such as sugars.

The results of the method for three other protein alignments previously used in [18] are available online (see “Data Availability” below).

## 4. Conclusions

In this work, a methodology is presented that goes one step beyond the current methods for detecting protein residues responsible for functional specificity (SDPs), by providing clues on the physicochemical basis of such specificity. Gaining insight into that physicochemical basis is important not only to understand the molecular mechanisms behind a protein’s function, but also to devise ways for modifying it in a more informed way.

Although a manual inspection of the amino-acid patterns of the SDP positions in the MSA could provide such information in simple cases, and indeed that was done in many SDP-related studies, a methodology that allows to do that in an automatic way is important for two main reasons. First, such a manual inspection could be unfeasible for large MSAs or non-trivial patterns of residue change, such as the well-studied position 37 of RasH discussed here. This position has been recognized as the main SDP for the Ras family and its involvement in controlling interaction specificity experimentally demonstrated. Nevertheless, the reason why these particular amino-acids are differentially conserved in the different subfamilies was not clear. According with our results, the reason could be related to the different propensities of these aminoacids to contribute to a beta-sheet that is fundamental for the interaction. These “subtle” patterns of chemical properties are not evident at first sight. Although many methods for detecting SDPs take into account, in one way or another, aminoacid similarities derived from substitution matrices, these matrices represent averages over many physicochemical properties, and some properties that might be important for certain functions in particular cases are not well reflected in such average values. Second, such a manual procedure can be applied to particular protein families only, while an automatic methodology allows processing large collections of MSAs, for example to obtain statistical results. Among other things, this methodology could be applied automatically to all available protein families to generate statistics on which physicochemical properties are more frequent in their SDPs, try to relate that with the families’ functions, etc. That would allow a large-scale analysis of the “functional toolkit” of the protein universe.

Related to that, in this work, we used 18 physicochemical properties (and factors), but in principle there is no limit in the number of amino-acid properties one can scan. There are repositories with hundreds of amino-acid quantitative scales, such as the Amino-Acid Index Resource [36] that can be incorporated in this method.

In this work, the method was applied to only two examples for which the results were discussed in detail (besides the other examples that were included as “blind” predictions). These examples, while covering the application of the method in two scenarios, are not enough for exhaustively benchmarking and quantifying its performance. That would require a large dataset with information not only on the residues responsible for functional specificity (SDPs), but on the physicochemical basis of those specificities as well. Such a resource is not available and, indeed, that lack of information on the physicochemical basis of functional specificity reinforces the need for methods such as that presented here.

## Figures and Tables

**Table 1:** Positions and physicochemical properties with highest SDP score in the MSA of RasH homologs. If the “property” column is empty, it means that the score is for the amino-acid substitution matrix.

Position	Residue	Property	Score
37	E		0.7569
37	E	betap	0.7538
37	E	pol_G	0.7355
37	E	flex	0.7331
37	E	hdf_KD	0.7311
73	R	factor5	0.7057
37	E	MW	0.7002
65	S	pol_G	0.6999
73	R	MW	0.6968
37	E	bulkiness	0.6879
66	A	hdf_KD	0.6862
73	R	betatLp	0.6861
73	R	coil	0.6860
73	R	alphap	0.6852
37	E	betatLp	0.6787
66	A	hdf_Cho	0.6760
73	R	factor3	0.6755
66	A	hdf_Eisenb	0.6745
71	Y	rel_mut	0.6734
66	A	factor1	0.6725
73	R	pol_G	0.6712
73	R	factor2	0.6710

**Table 2:** Positions and physicochemical properties with highest SDP score in the MSA of RasH structural homologs. If the “property” column is empty, it means that the score is for the amino-acid substitution matrix.

Position	Residue	Property	Score
16	K	pol_G	0.6753
10	G	betap	0.6597
16	K		0.6493
10	G	factor5	0.6098
76	E	hdf_Eisenb	0.6025
76	E	hdf_KD	0.5960
10	G	hdf_KD	0.5932
76	E	factor1	0.5929
10	G	pol_G	0.5825
76	E		0.5755
10	G	bulkiness	0.5637
16	K	betap	0.5614
16	K	flex	0.5555
10	G		0.5481
13	G	betap	0.5446
76	E	hdf_Cho	0.5421
76	E	rel_mut	0.5356
115	G	factor1	0.5283
89	S	factor4	0.5168
10	G	hdf_Eisenb	0.5095
10	G	betatLp	0.5052
10	G	factor1	0.5043

## Data Availability

All datasets and software used in this study are available upon request. Additional results of the method for other families of proteins are available at: http://csbg.cnb.csic.es/pazos/Xdet/propdet/ (accessed on 25 November 2021).
